# Nitric Oxide Resistance Reduces Arteriovenous Fistula Maturation in Chronic Kidney Disease in Rats

**DOI:** 10.1371/journal.pone.0146212

**Published:** 2016-01-04

**Authors:** Irma L. Geenen, Felix F. Kolk, Daniel G. Molin, Allard Wagenaar, Mathijs G. Compeer, Jan H. Tordoir, Geert W. Schurink, Jo G. De Mey, Mark J. Post

**Affiliations:** 1 Department of Physiology, Cardiovascular Research Institute Maastricht, Maastricht University Medical Center, Maastricht, The Netherlands; 2 Department of General Surgery, Cardiovascular Research Institute Maastricht, Maastricht University Medical Center, Maastricht, The Netherlands; 3 Department of Pharmacology, Cardiovascular Research Institute Maastricht, Maastricht University Medical Center, Maastricht, The Netherlands; Albany Medical College, UNITED STATES

## Abstract

**Background:**

Autologous arteriovenous (AV) fistulas are the first choice for vascular access but have a high risk of non-maturation due to insufficient vessel adaptation, a process dependent on nitric oxide (NO)-signaling. Chronic kidney disease (CKD) is associated with oxidative stress that can disturb NO-signaling. Here, we evaluated the influence of CKD on AV fistula maturation and NO-signaling.

**Methods:**

CKD was established in rats by a 5/6^th^ nephrectomy and after 6 weeks, an AV fistula was created between the carotid artery and jugular vein, which was followed up at 3 weeks with ultrasound and flow assessments. Vessel wall histology was assessed afterwards and vasoreactivity of carotid arteries was studied in a wire myograph. The soluble guanylate cyclase (sGC) activator BAY 60–2770 was administered daily to CKD animals for 3 weeks to enhance fistula maturation.

**Results:**

CKD animals showed lower flow rates, smaller fistula diameters and increased oxidative stress levels in the vessel wall. Endothelium-dependent relaxation was comparable but vasorelaxation after sodium nitroprusside was diminished in CKD vessels, indicating NO resistance of the NO-receptor sGC. This was confirmed by stimulation with BAY 60–2770 resulting in increased vasorelaxation in CKD vessels. Oral administration of BAY 60–2770 to CKD animals induced larger fistula diameters, however; flow was not significantly different from vehicle-treated CKD animals.

**Conclusions:**

CKD induces oxidative stress resulting in NO resistance that can hamper AV fistula maturation. sGC activators like BAY 60–2770 could offer therapeutic potential to increase AV fistula maturation.

## Introduction

The prevalence of end-stage renal disease (ESRD) has increased during the last decade and amounted over 500.000 patients in the United States in 2009.[1] The majority of patients rely on hemodialysis, and for them a functional vascular access is essential. According to the National Kidney Foundation Kidney Disease Outcomes Quality Initiative and the European Best Practices Guidelines for vascular access, first choice for vascular access is an autologous arteriovenous (AV) fistula.[2, 3] However, at short term, AV fistulas show rather high primary failure rates due to non-maturation. Non-maturation occurs in 28–53% of the radiocephalic fistulas, with higher incidence in older, female patients and patients with extensive vascular disease.[4, 5] According to the Dialysis Access Consortium, maturation of an AV fistula is defined as the ability to use the fistula for dialysis within 4 months after fistula creation, and a minimum flow of 300mL/min for at least 8 dialysis sessions during the ensuing 30 days.[6]

The key requirement for AV fistula maturation is dilation of the arterial and venous vessel segments, to generate a sufficient flow required for hemodialysis and to prevent thrombosis.[7] Creation of an arterial-venous anastomosis leads to a low-resistance circuit and as a result of this, blood flow through this segment will increase. Increased flow will raise shear stress that stimulates endothelial cells (ECs) to synthesize nitric oxide (NO) that induces vessel dilation via cyclic guanosine monophosphate (cGMP) signaling.[8–10] NO binds to its cognate receptor soluble guanylate cyclase (sGC) in the smooth muscle cell (SMC), facilitating the conversion of guanosine triphosphate (GTP) into the second messenger cGMP. cGMP stimulates SMC relaxation via protein kinase G (PKG) activation followed by a decrease in intracellular calcium levels.[11–13]

It has been hypothesized that one of the key events in AV fistula maturation failure is endothelial dysfunction[7, 14] as a result of uremia-induced oxidative stress.[15–18] Endothelial dysfunction in uremia is characterized by diminished NO bioavailability due to decreased endothelial NO synthase (eNOS) expression[19], reduced tetrahydrobiopterin (BH4) levels resulting in eNOS uncoupling[20, 21], high amounts of the endogenous eNOS-inhibitor asymmetric dimethylarginine (ADMA)[22] and scavenging of NO by free radicals.[18]

Previous reports on the influence of chronic kidney disease (CKD) on AV fistula function showed increased neointima formation due to higher cell-turnover in CKD[23] and an enhanced migratory phenotype of SMCs.[24] However, the influence of CKD on NO-signaling and AV fistula maturation has not been investigated yet.

Therefore, we aimed to investigate the influence of CKD on endothelial and SMC function in relation to AV fistula maturation. Nephrectomized and control rats underwent AV fistula creation and endothelial and SMC function were studied ex vivo in vessel rings in a wire myograph. NO-signaling was assessed by means of an oxidator of sGC, the sGC stimulator BAY 41–2272 and sGC activator BAY 60–2770. The latter was also administered to CKD animals in order to enhance AV fistula maturation.

## Subjects and Methods

### Animals

Experimental protocols were approved by the Dier Ethische Commissie Maastricht University, Maastricht, The Netherlands, approval number: DEC 2010–044 and were conducted according to international guidelines (American Physiological Society Guiding Principles for the Care and Use of Vertebrate Animals in Research and Training). Wistar rats weighting 275–325 grams, 9–11 weeks old, were purchased from Harlan Laboratories (Horst, The Netherlands). Animals were housed in normal cages with free access to water and standard chow diet and were kept in climate-controlled rooms (21°C and 55% relative humidity) with a 12h cycle of light and darkness. All surgical procedures were carried out under general anesthesia with isoflurane 2% combined with an analgesic (buprenorphin 0.03 mg/kg) under sterile conditions in the laboratory during daytime. Allocation to experimental groups was performed in a one-by-one sequential manner. Weight loss >20% or lethargic behavior after 5/6th nephrectomy was defined as a humane endpoint.

Rats underwent 5/6^th^ nephrectomy as described previously[25] by removing the right kidney and ligating two of the three main arterial branches of the left kidney. Care was taken to ensure that two third of the kidney appeared ischemic, if necessary an additional branch was ligated. In sham animals, the abdomen was opened and kidneys were exposed but left intact.

Blood pressure was measured with a tail-cuff system (CODA™, Kent Scientific Corporation, Torrington, CT, USA) before AV fistula surgery and before euthanasia. To prevent stress-effects, animals were trained before measurements. At least 5 accurate measurements per animal were used for analysis of diastolic, mean and systolic pressure.

### AV fistula creation

**Fig 1:**
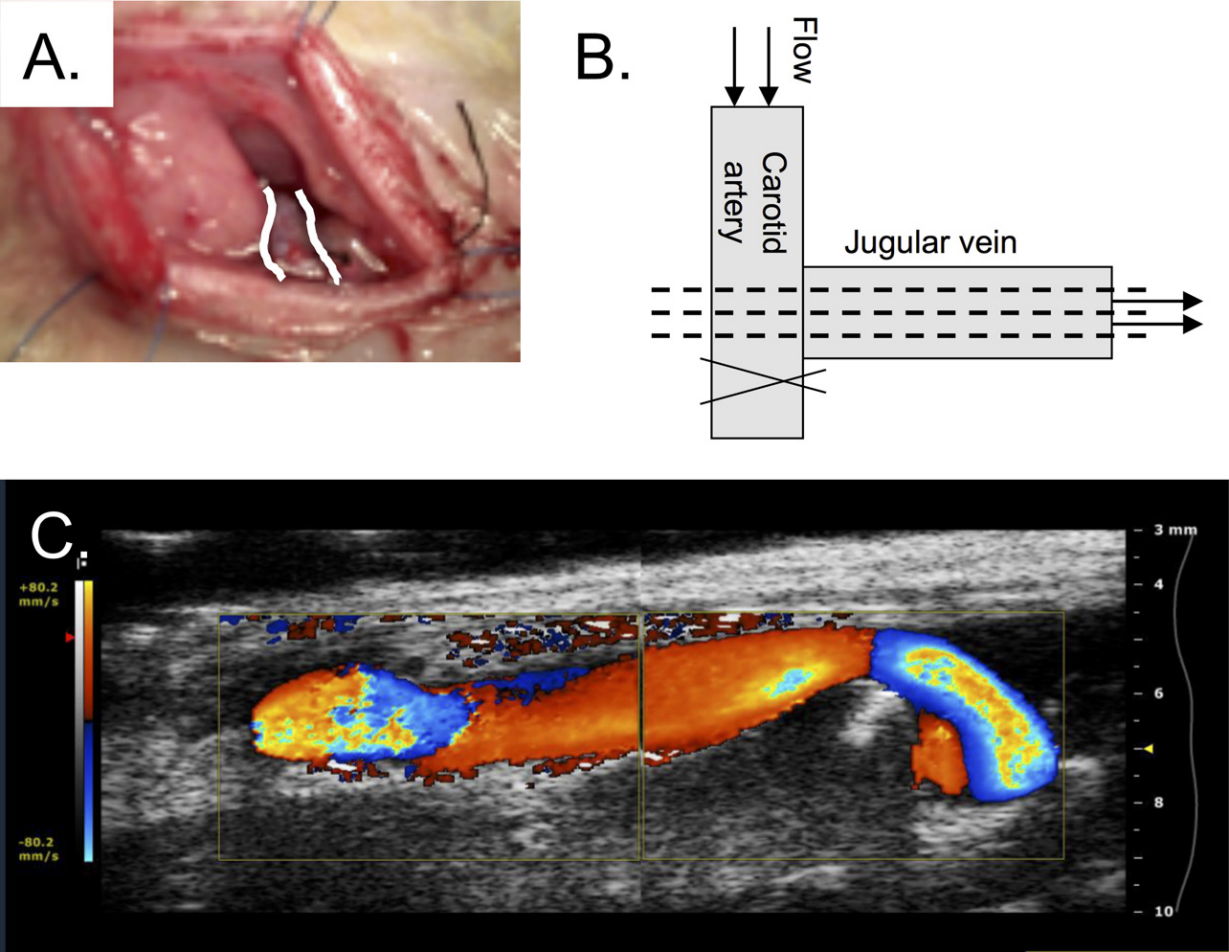
AV fistula. A. AV fistula created by anastomosing the jugular vein to the carotid artery in an end-to-side manner. The venous part is depicted with white lines. Due to its anatomical, deeper location, the carotid artery cannot be observed in this picture. B. For the purpose of (immuno)histochemistry longitudinal sections were cut through the AV fistula (dashed lines). C. Representative color Doppler ultrasound image of AV fistula (distal part at the left side).

Six weeks after 5/6^th^ nephrectomy or sham procedure, an AV fistula of the jugular vein and the carotid artery was created (Fig 1A). After opening of the skin, the jugular vein was exposed and an end-to-side anastomosis was created to the ipsilateral carotid artery with eight interrupted sutures of 10–0 monofilament (Monosof™, Covidien, Mansfield, MA, USA). The distal part of the carotid artery was ligated.

### AV fistula follow-up

**Fig 2:**
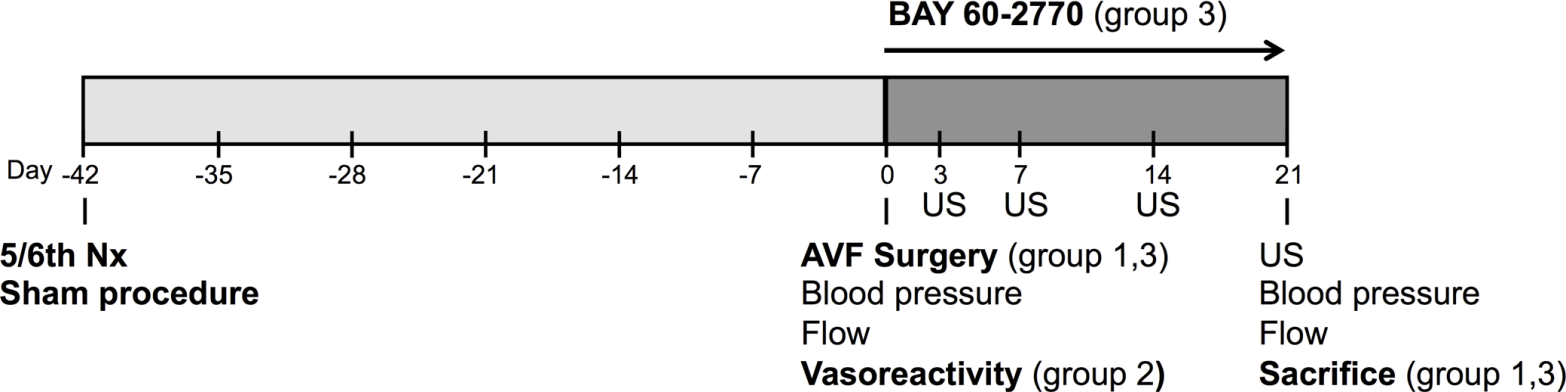
Study design. Animals underwent a 5/6^th^ nephrectomy or sham procedure. After six weeks, an AV fistula was created. Group 2 was euthanized after these six weeks for measurements of vasoreactivity. AV fistula animals (group 1,3) were monitored for another 21 days with ultrasound measurements conducted after 3, 7, 14 and 21 days. Hereafter, animals were euthanized after blood pressure and flow assessment. Group 3 was treated with BAY 60–2770 or vehicle after AV fistula surgery and underwent similar follow-up as animals in group 1.

Immediately after AV fistula creation and three weeks later, flow through the arterialized vein was measured with an ultrasonic flow probe (Transonic Systems BV, Maastricht, The Netherlands). Mean diameter of the AV fistula was determined with ultrasound measurements in anesthetized animals (isoflurane 2.8%). Measurements were conducted at 3, 7, 14 and 21 days after AV fistula creation with the VEVO 770 and a 30 mHz transducer (VisualSonics Inc., Toronto, Canada) (Fig 2). B-mode images were obtained from the proximal carotid artery and the arterialized vein.

After three weeks of follow-up (Fig 2), animals were euthanized under anesthesia by means of aortic puncture and complete blood removal. Serum was collected for creatinine and urea assessment. Post-mortem, animals were flushed with saline and perfusion-fixed with 2% formalin at 100 mmHg pressure to preserve vessel morphology. The AV fistula was removed en bloc, including a short proximal arterial part, connected to the longer arterialized vein and a short part of the distal ligated artery. One can imagine this a certain 'T-shaped' specimen (Fig 1B). These were stored in formalin overnight and then transferred to ethanol 70% for later paraffin embedding. Heart weight and left kidney weight were recorded. Serum analyses were performed in the clinical laboratory by autoanalyzer techniques.

### Treatment with BAY 60–2770

The effect of BAY 60–2770 on AV fistula maturation was studied in CKD animals. After creation of the AV fistula, animals were treated with BAY 60–2770 (0.3 mg/Kg) or vehicle for 3 weeks until euthanasia. BAY 60–2770 was dissolved in the vehicle consisting of transcutol 10%, cremophor 20% and H2O 70% and was administered by daily oral gavage.[26] Plasma levels of BAY 60–2770 were determined in plasma by high-pressure liquid chromatography (HPLC) coupled with a tandem mass spectrometer (MS/MS) (MDS Sciex API 4000; Applied Biosystems, Foster City California). Samples were collected from CKD and control animals at 30, 60 and 120 minutes after oral administration.

### Histology and morphometry

During the fixation and paraffinization process the fistulas were carefully flattened, which enabled us to cut longitudinal sections through the anastomosis and distal arterialized vein. Serial sections of 5 μm thickness were obtained every 120 μm throughout the AV anastomosis as depicted in Fig 1B. Hematoxylin and eosin staining was performed with (Mayer’s Hematoxyline and Eosine Y (0.2%) (Klinipath, Duiven, The Netherlands) and Elastin von Gieson (EvG) staining was performed according to the manufacturer’s protocol (Merck, Darmstadt, Germany). Sections were stained for SMCs with mouse monoclonal anti-αSMA (A2547, Sigma, Zwijndrecht, The Netherlands) (1:3000, overnight), for oxidative stress with rabbit polyclonal anti-nitrotyrosine and ECs were stained by a mouse monoclonal CD31 antibody (1:100, overnight) (AbD Serotec, Puchheim, Germany). After incubation with the appropriate secondary antibody, they were subjected to avidin-biotin horseradish peroxidase-DAB procedure (Vector Labs, Burlingame, CA, USA and DakoCytomation, Dako Netherlands BV, Heverlee, Belgium, respectively) and counterstained with haematoxylin (Klinipath, Duiven, the Netherlands). To determine the amount of intimal hyperplasia, EvG sections were photographed with a Leica DMI 300B (Rijswijk, The Netherlands). Morphometric analysis for intima and media thickness was automated (Qwin V3, Leica) and performed in three sections of the AV fistula on two high-power fields per section (Fig 1B). Average thickness was calculated by dividing surface area by the length of the vessel segment. Amount of DAB-stained pixels in anti-nitrotyrosine stained sections was quantified by automatic analysis in Image-Pro Plus (MediaCybernetics, Rockville, MD, USA). The area of interest (AOI) was acquired based on the total amount of stained tissue per high-power field section. Then, color intensity of DAB-stained pixels was identified and percentage of DAB-stained pixels per total amount of pixels in the AOI was automatically calculated. For analysis of endothelial coverage we used a semi-quantitative analysis using the following scores per section (arterial/venous part): 1 = No ECs visible, 2 = Focal ECs, 3 = Diffuse presence of ECs and 4 = Complete endothelial coverage. Frequencies of scores were calculated per group. The analysis was blinded to treatment and experimental group.

### Isometric tension measurements

Six weeks after 5/6^th^ nephrectomy or sham procedure, animals were euthanized by inducing hypovolemia via aortic puncture. The carotid arteries were carefully dissected without damaging the endothelium and 2mm long segments were mounted in a wire myograph (DMT, Aarhus, Denmark). During contraction with phenylephrine (10μM) endothelial-dependent relaxation was studied by means of stimulation with acetylcholine (ACh,10^−10^–10^-5^M, both Sigma). Blocking of NOS with L-NAME (10μM, Sigma) was also performed to control for non-NO mediated effects of ACh. The NO-donor sodium nitroprusside (SNP,10^−10^–10^-5^M, Sigma) was used to evaluate the relaxation capacity of SMCs. BAY 41–2272, a stimulator of sGC (Bayer, Leverkusen, Germany) and BAY 60–2770, an activator of oxidized sGC (Bayer) (both kindly provided by Dr. J.P. Stasch, Bayer) were used to evaluate the oxidative state of sGC. 1H-[1,2,4]oxadiazolo[4,3-a]quinoxalin-1-one (ODQ, Sigma) was used to actively oxidize sGC in combination with the BAY compounds.

### Western Blots

Using complete radio-immunoprecipitation assay lysis buffer (Santa-Cruz Technology, Santa Cruz, USA), with additional proteases and phosphatases inhibitors (Thermo Fisher, Aalst, Belgium), vessels were boiled at 95°C for 10 minutes. The partially lysed vessels were transferred to a BeadBeater (Biospec Products, Barlesville, USA) and beated for 2x30 seconds and cooled on ice in between. Protein concentration was determined using the bicinchoninic acid method (Pierce, Rockford, USA) and mixed with 4x sample loading buffer (BioRad, Veenendaal, The Netherlands), 20x reducing agent (BioRad) before boiling for 5 minutes at 95°C. Samples were cooled down to RT, centrifuged for 1 minute at 13,400 g and loaded on a gel (4–12% Bis-Tris, BioRad). The gel was blotted on a polyvinylidene difluoride membrane (BioRad) overnight 30V at 4°C. The blotting membrane was blocked by incubation with 3% milk powder in TBS-Tween and incubated with a primary antibody against sGC or β-actin (Abcam, Cambridge, UK and Sigma). Next, the blot was incubated with a peroxidase conjugated secondary antibody and bands were detected using the SuperSignal West Femto substrate (Pierce, Thermo Scientific, Rockford, IL, USA). Visualization was performed using the ChemiDoc XRS system (BioRad).

### Statistical analysis

All data are expressed as mean ± SEM. Contractile responses are depicted as percentage of relaxation after phenylephrine precontraction. Curves were fitted and data were analyzed with Prism 5.0c for Mac (GraphPad Software, San Diego, CA, USA) and Microsoft Excel for Mac 2011. Groups were compared using Student’s T-test and two-way ANOVA repeated measured for time-bound analysis. *P-*values <0.05 were considered significant.

## Results

### Effects of 5/6^th^ nephrectomy

Animals were exposed to CKD (n = 10) for six weeks before AV fistula creation. Control animals were submitted to a sham operation (*n* = 11). Serum creatinine (85±12.5 vs. 33±1.5 μmol/L, *P*<0.05) and urea (17.9±2.8 vs. 7.5±0.5 mmol/L, *P*<0.05) were significantly higher in CKD animals compared to controls. Systolic blood pressure was elevated in CKD animals before and after AV fistula creation. Interestingly, heart weight was also higher in CKD animals (2.51 ± 0.28 vs. 2.14±0.31 gram, *P*<0.05) (Table 1). 33% of animals reached the humane endpoints of excessive weight loss and/or lethargy (>20%) as a result of CKD and were euthanized. Stroke occurred in one animal after fistula surgery, probably due to a cerebral embolus.

**Table 1:** Animal characteristics.

		Control (*n* = 11)	CKD (*n* = 10)
Weight (g)		421 ± 14	395 ± 13
Serum creatinine (μmol/L)		33 ± 1.5	85 ± 12.5^**^
Serum urea (mmol/L)		7.5 ± 0.5	17.9 ± 2.8^**^
Blood pressure (mmHg)			
Diastolic	t = 42	105 ± 7	129 ± 14
	t = 63	89 ± 3	94 ± 5
Systolic	t = 42	147 ± 8	174 ± 12^*^
	t = 63	130 ± 4	146 ± 5^*^
Mean	t = 42	118 ± 7	144 ± 14
	t = 63	102 ± 4	111 ± 5
Heart weight (mg)		2.14 ± 0.11	2.51 ± 0.09^*^

^*^P<0.05

^**^P<0.01 compared to control

### CKD animals have diminished AV-fistula flow

**Fig 3:**
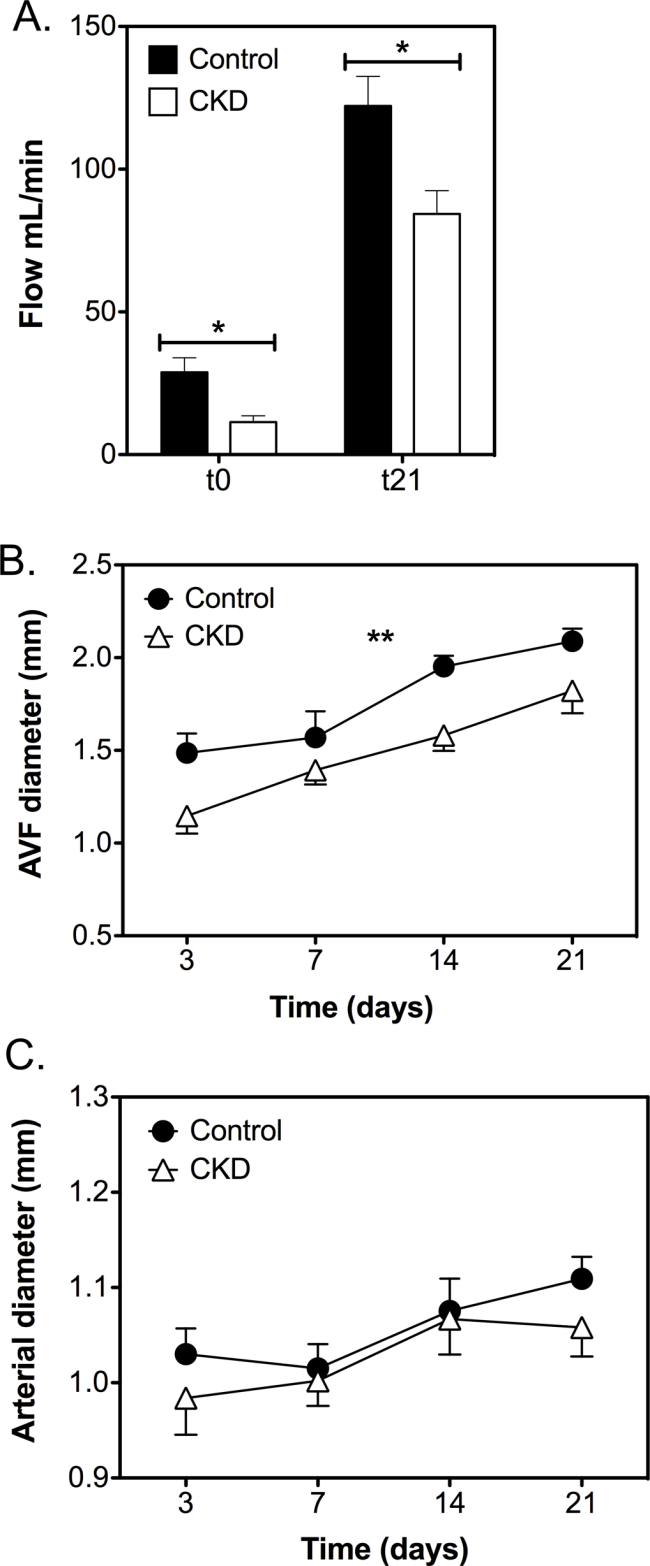
AV fistula function. A. AV fistula flow increased over time and was significantly decreased in CKD (*n* = 10) animals compared with controls (*n* = 11) at both time points. B. Ultrasound measurements at different time points revealed an increase in diameter over time. AV fistula diameter was significantly larger in controls (*n* = 11) compared with CKD animals (*n* = 10). C. Diameters of the proximal arterial segment increased over time (*P*<0.001) but were not significantly different between groups. Data are expressed as mean ± SEM. *P<0.05, **P<0.01.

Blood flow through the AV fistula was measured directly after AV fistula creation and three weeks post-operatively. Flow increased over time for both groups, but was significantly lower in CKD animals compared to controls at both time points (t0: 11.4±2.2 vs. 28.8±5.1 mL/min, and t21: 84±8 vs. 122±10 mL/min, *P*<0.05; Fig 3A). Vessel diameters increased over time for both groups and were lower in CKD animals (*P*<0.01; Fig 3B). Diameters of the proximal arterial segment increased over time (*P*<0.001) but were not significantly different between groups (Fig 3C).

### Increased oxidative stress in AV fistulas of CKD animals

**Fig 4:**
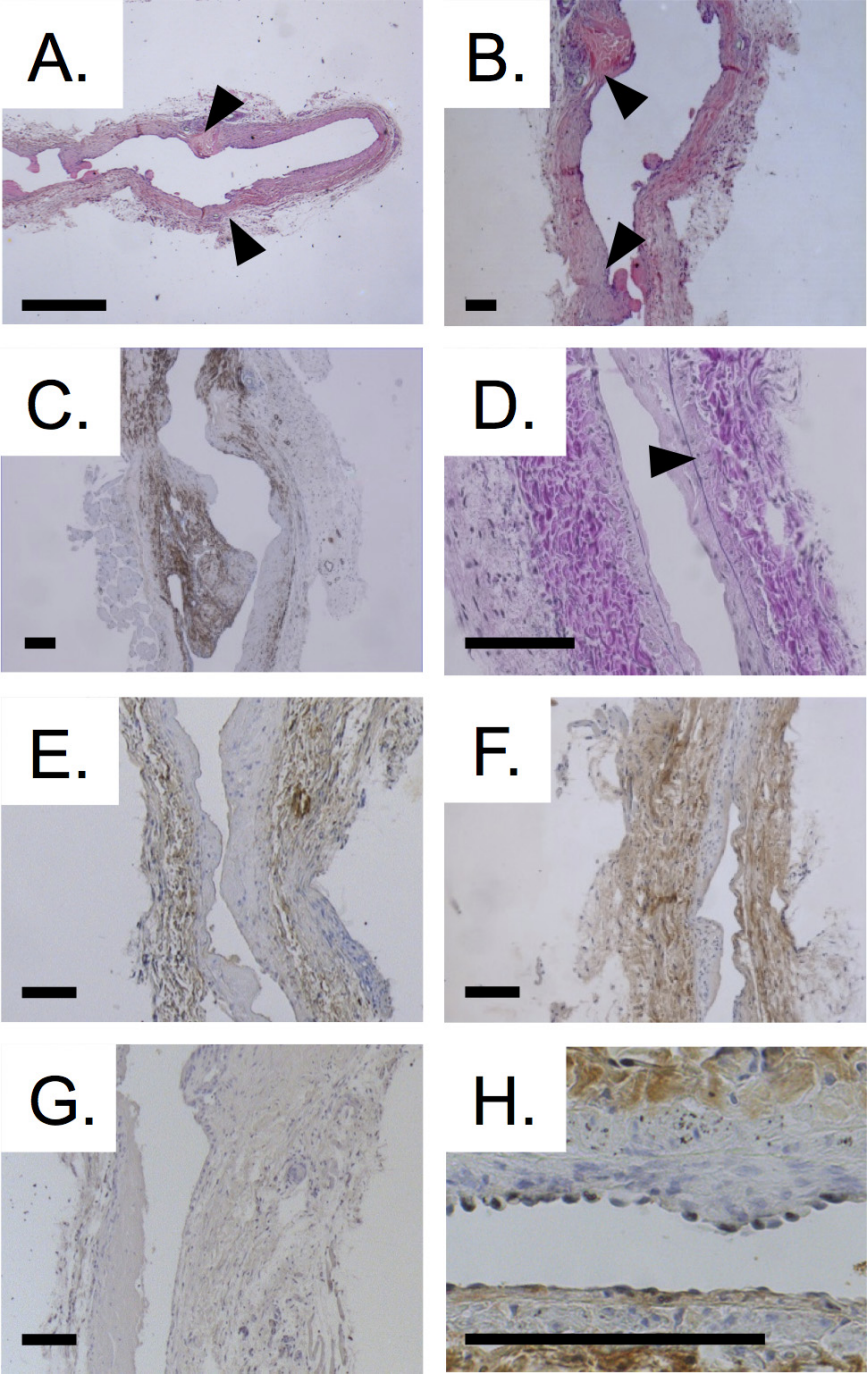
Histology of AV fistulas. A. HE staining of AV fistula. Arrowheads indicate anastomosis. Arterial part located on the right side. B. HE staining showed intima hyperplasia (arrowheads) close to the anastomosis site as well as more distal in the venous outflow tract. C. Staining for αSMA demonstrated that SMCs are the most abundant cell type in the intima hyperplasia lesions. D. Determination of intima and media thickness was based on EvG stained sections using the internal and external elastic membrane as boundaries (arrowheads). E. Oxidative stress was displayed by staining for nitrotyrosine. Oxidative stress was most prominent in the media and adventitia. AV fistula from control animal. F. Substantial amount of nitrotyrosine in AV fistula from CKD animal. G. Negative control for anti-nitrotyrosine staining. H. Staining for CD31 revealed absence of ECs on intimal hyperplasia region (upper side) and presence of ECs on non-affected vessel wall (lower side). Scale bars represent 100 μm.

HE-stained sections of AV fistulas showed areas of intima hyperplasia close to the anastomosis and also more distal in the venous outflow tract (Fig 4A and 4B). Immunohistochemical staining for αSMA cells indicated that SMCs were the most abundant cells in the intima hyperplasia lesions (Fig 4C).

Vessel morphometry was determined in EvG stained sections. Neither intima and media thickness, nor their ratio differed between controls and CKD animals (measured in 3 sections per animal, Table 2, Fig 4D).

**Table 2:** Histological analysis AV fistulas.

	Intima thickness (mm)	Media thickness (mm)	Intima/media ratio	Oxidative stress (% DAB positive pixels)
**Control**	25.7 ± 5.1.0	46.8 ± 3.9	0.63 ±0.15	13.7 ± 2.9
**CKD**	29.2 ± 4.0	46.9 ± 3.0	0.65 ±0.09	28.4 ± 6.1^*^

^*^P<0.05 compared to control

**Fig 5:**
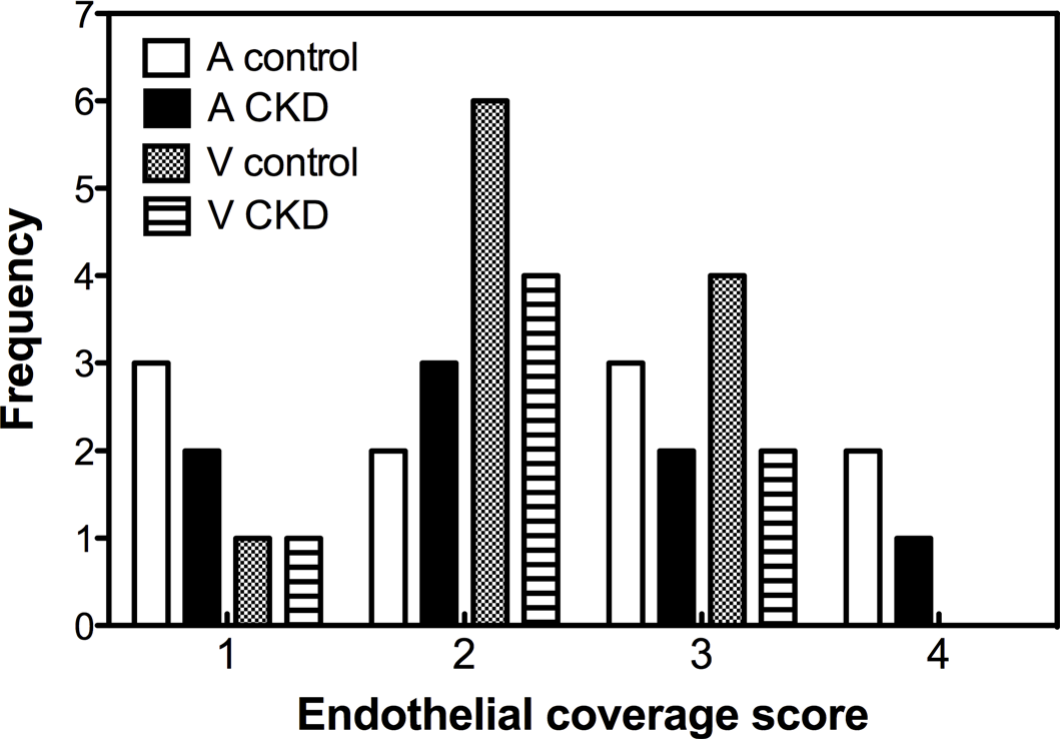
Endothelial coverage. Frequency distribution of endothelial coverage scores, assessed in a semi-quantitative manner. A = artery, V = venous part of AV fistula. 1 = No ECs visible. 2 = Focal presence of ECs. 3 = Diffuse presence of ECs. 4 = Complete coverage of luminal side by ECs.

Presence of nitrotyrosine indicating oxidative stress was most prominent in the media and adventitia. AV fistulas from CKD animals showed higher levels of nitrotyrosine indicating increased oxidative stress (Table 2, Fig 4E, 4F and 4G). Endothelial coverage was assessed in a semi-quantitative manner in CD31 stained sections (Fig 4H, Fig 5). Areas devoid of ECs could be observed in the arterial part and arterialized vein in particular on the luminal side of intima hyperplasia lesions (Fig 4H). No significant differences were found between endothelial coverage scores of control and CKD animals.

### CKD leads to NO resistance

**Fig 6:**
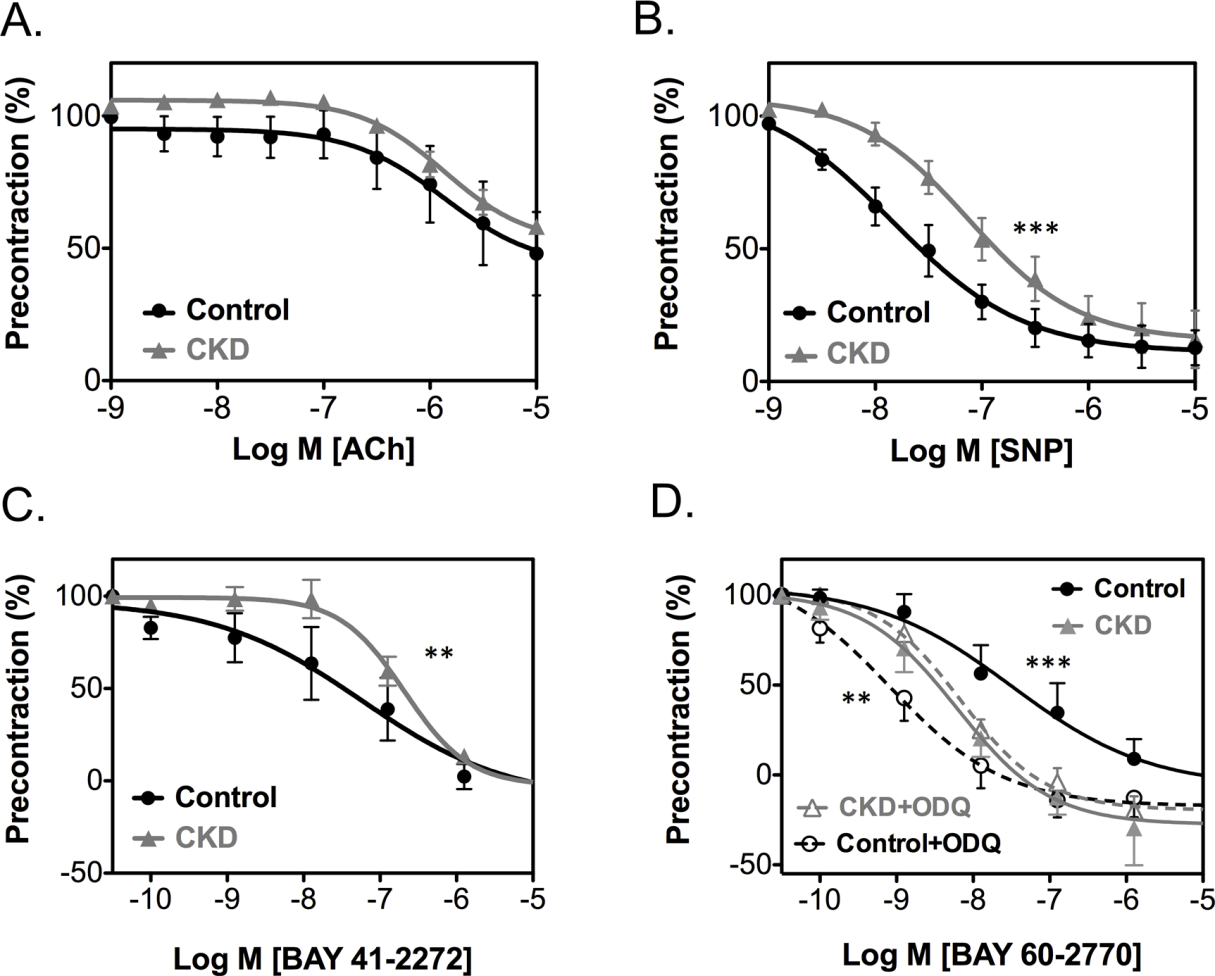
Vasoreactivity of arteries from CKD animals and controls. Endothelium-dependent relaxation after ACh was comparable for CKD and controls (A). Stimulation with SNP, which resembles endothelium-independent relaxation, (B) and heme-dependent BAY 41–2272 (C) resulted in decreased relaxation for the CKD group compared to controls. Relaxation induced by heme-independent BAY 60–2770 caused an augmented response in CKD animals (D, filled symbols). After artificial oxidation, BAY 60–2770 stimulation resulted in increased relaxation of control vessels (D, open symbols). Data are expressed as mean ± SEM. *n* = 6 at least. **P<0.001, ***P<0.0001

Vasomotor responses of carotid arteries from CKD animals and controls were recorded in a wire myograph after six weeks of CKD. Vessel diameters and wall tension after precontraction with phenylephrine were not different between groups (data not shown). Endothelium-dependent relaxation did not differ between CKD and controls (Fig 6A) indicating normal endothelial function. Blocking of eNOS by L-NAME abrogated ACh-induced relaxation (data not shown), confirming that endothelium-dependent relaxation was completely attributable to NO-directed vasodilation. In contrast, vessel relaxation in response to the exogenous NO-donor SNP (endothelium-independent relaxation) was decreased in the CKD group (LogIC50: -7.83±0.29M vs. -7.13±0.16M *P*<0.0001; Fig 6B), which suggests resistance for NO of the NO-receptor sGC.

NO resistance of sGC was further characterized using BAY 41–2272 and BAY 60–2770. Sensitivity for BAY 41–2272, a stimulator of sGC when heme is present in its native state, was decreased in CKD animals (LogIC50: -6.82±0.08M vs. -7.21±0.41M, *P*<0.001; Fig 6C) supporting NO resistance of sGC. In contrast, stimulation with the heme-independent sGC activator BAY 60–2770 showed higher sensitivity in CKD animals (LogIC50: -8.35±0.23M vs. -7.62±0.34M, *P<*0.0001; Fig 6D). Together, these data indicate that the heme group of sGC in CKD animals is oxidized or reduced, which leads to inability of sGC to be activated by NO or BAY 41–2272.

**Fig 7:**
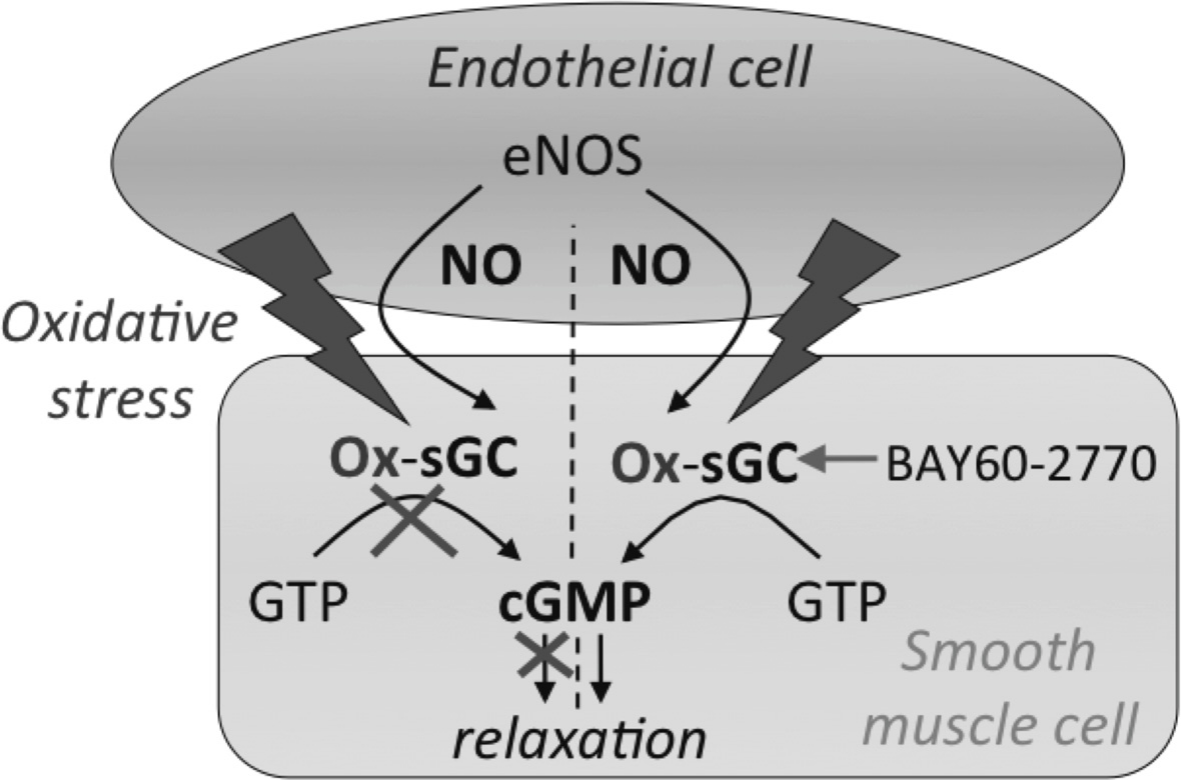
Nitric oxide (NO)-resistance in the vessel wall. Oxidative stress can oxidize the NO-receptor in smooth muscle cells (SMCs), soluble guanylate cyclase (sGC), leading to an altered redox state and/or loss of the heme group. After NO stimulation, guanosine triphosphate (GTP) is no longer converted into cyclic guanosine monophosphate (cGMP), and relaxation of SMCs, usually a result of increased cGMP levels that activate protein kinases thereby decreasing intracellular calcium, will no longer take place. BAY60-2770 is able to activate oxidized sGC thereby restoring vascular relaxation.

Using ODQ the sGC heme group was actively oxidized, after which vessel rings were stimulated with BAY 60–2770. This resulted in an increased sensitivity of control vessels compared to CKD arteries for BAY 60–2770 (LogIC50: -9.10±0.38M vs. -8.33±0.12M, *P<*0.001; Fig 6D) suggesting that sGC is oxidized in CKD (Fig 7).

### sGC expression is decreased in CKD animals

**Fig 8:**
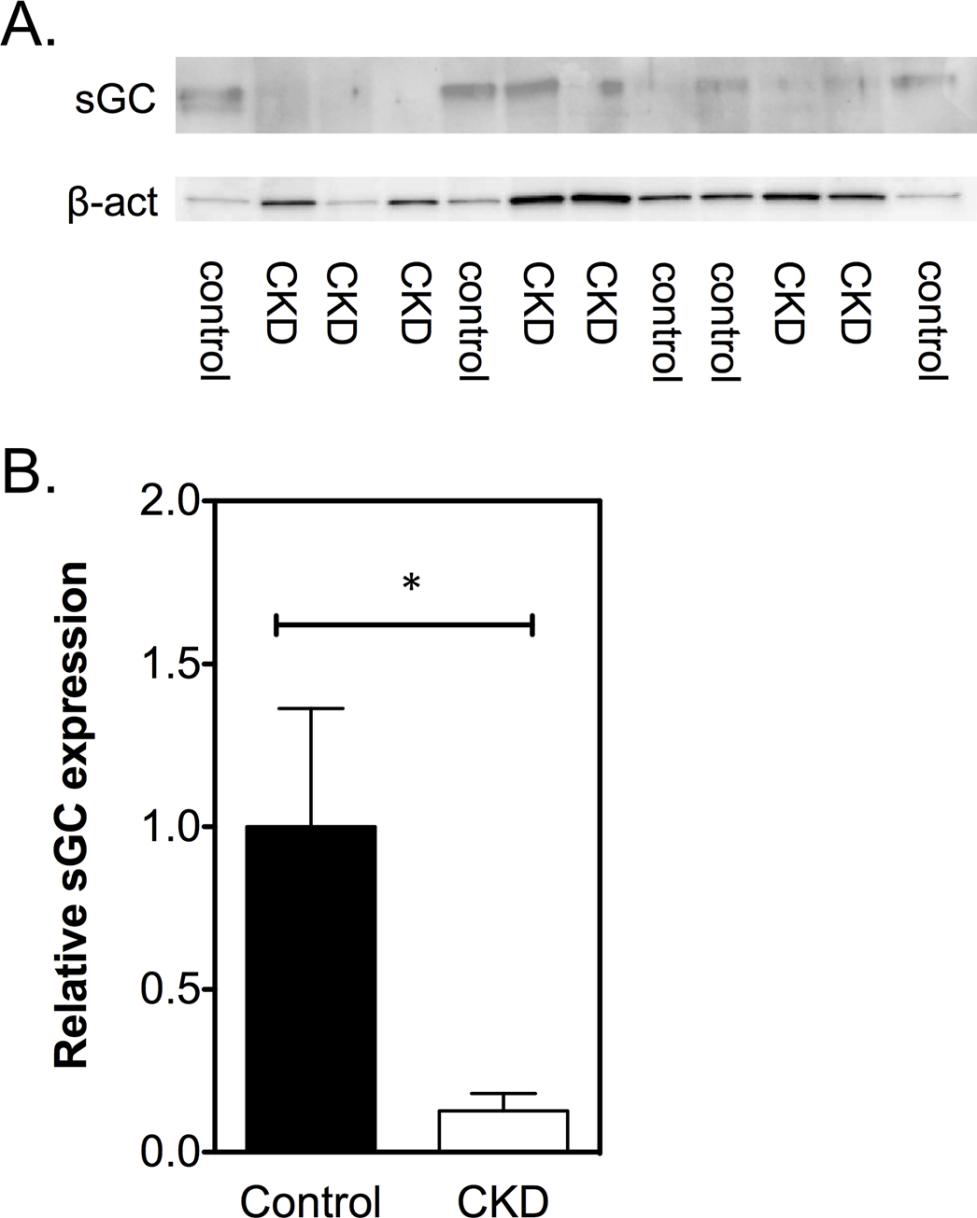
Protein expression of sGC. A. Expression was calculated using BioRad Quanty One and corrected for input using β-actin. B. sGC was significantly lower expressed in CKD arteries (*n* = 7) compared to controls (*n* = 6). Controls were used as reference and set at 1.0. Data are expressed as mean ± SEM. *P<0.05

After sGC oxidation with ODQ, control vessels turned out to be more sensitive to BAY 60–2770 compared to CKD vessels, which could be related to a decrease in sGC in CKD. Therefore, sGC expression was analyzed by means of Western Blot. Indeed, arteries from CKD animals expressed significantly less sGC than control animals (0.13±0.05 vs 1.00±0.36, *P<*0.05; Fig 8A and 8B).

### BAY 60–2770 increases AV fistula diameter in CKD animals

**Fig 9:**
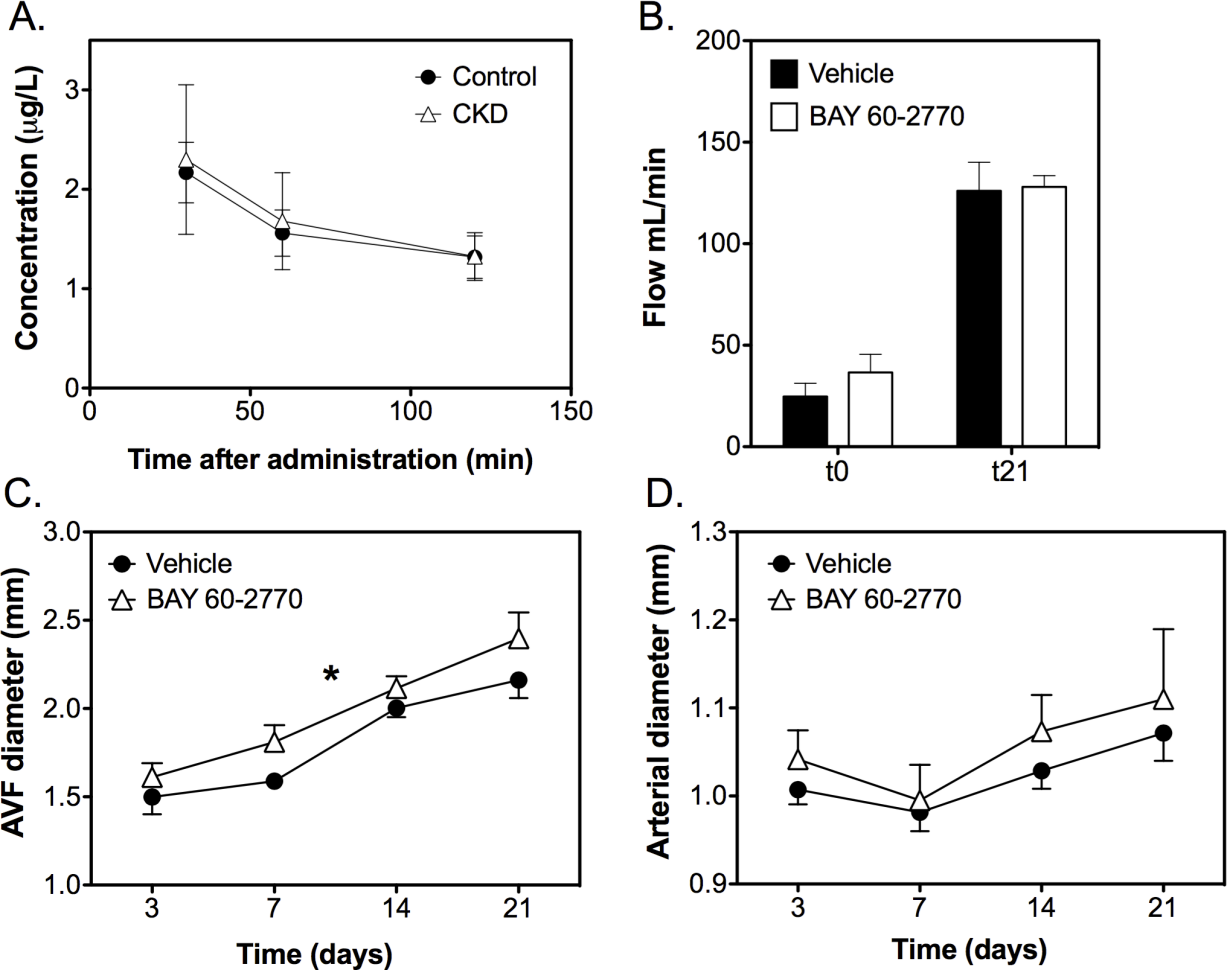
Influence of treatment with BAY 60–2770 on AV fistula function. A. Plasma levels of BAY 60–2770 at several time points after administration did not differ between CKD (*n* = 5) and control animals (*n* = 4). B. Flow increased over time and did not differ between groups. C BAY 60–2770 (*n* = 6) increased AV fistula diameter compared to vehicle treated animals (*n* = 7). D. Proximal arterial diameters increased over time (*P*<0.05) but were not significantly different between groups. Data are expressed as mean ± SEM. *P<0.05.

To increase SMC relaxation and thus AV fistula flow, BAY 60–2770 was administered orally to CKD animals after creation of an AV fistula. As shown in Fig 9A, BAY 60–2770 was successfully absorbed and distributed in the plasma. No differences were observed between groups. Creatinine and urea levels were not affected by BAY60-2770. Chronic treatment with BAY 60–2770 did not significantly modify blood pressure (mean blood pressure t0 vehicle vs. BAY 60–2770: 148±4 vs. 162±11 mmHg; mean t21 vehicle vs. BAY 60–2770: 125±8 vs. 131±12 mmHg). Diameters of AV fistulas of BAY 60–2770 treated animals were larger compared to the vehicle group (*P<*0.05; Fig 9C). However, this increment in diameter did not result in increased flow; with flow at both time points (t0 and t21) being not significantly different between groups (t0 vehicle vs. BAY 60–2770: 25±6 vs. 37±9 mL/min; t21 vehicle vs. BAY 60–2770, 126±14 vs. 128±6 mL/min; Fig 9B). Diameters of the proximal arterial segment increased over time (*P*<0.05) but were not significantly different between groups (Fig 9D).

The effect of BAY 60–2770 on vascular wall structure was determined in EvG stained sections. No effects on intima and media thickness were observed, and levels of oxidative stress were also not affected by BAY 60–2770 (Table 3).

**Table 3:** Histological analysis AV fistulas.

	Intima thickness (mm)	Media thickness (mm)	Intima/media ratio	Oxidative stress (% DAB positive pixels)
**Vehicle**	22.7 ± 5.7	49.3 ± 4.1	0.53 ± 0.15	32.0 ± 11.8
**BAY 60–2770**	18.5 ± 5.1	44.4 ± 7.4	0.44 ± 0.14	21.1 ± 8.6

## Discussion

Despite the fact that much research has addressed neointima formation and its relation to AV fistula failure in the long-term, early failure as a result of non-maturation has not been investigated extensively. In this study, we investigated the relationship between CKD, NO-signaling and early AV fistula function. We show that the uremic state of CKD was associated with decreased AV fistula maturation, as hypothesized by several authors.[7, 14, 27] This is probably related to increased oxidative stress levels in the vessel wall of uremic animals, as shown by higher nitrotyrosine levels. Oxidative stress can lead to endothelial dysfunction[28, 29] and diminished NO-signaling by means of superoxide production and NO depletion by eNOS uncoupling.[19, 20, 22, 30] To evaluate NO-signaling in CKD we studied endothelial and SMC function by assessing vasoreactivity in a wire myograph. Hereby, we demonstrated for the first time a different mechanism for disturbed NO signaling in CKD, based on NO resistance of arterial smooth muscle. Isolated carotid arteries of CKD-animals showed normal vasorelaxing responses to ACh but diminished relaxation in response to SNP indicating disturbed SMC-function. This could be ascribed to NO resistance of the NO-receptor sGC as shown by an increased vasorelaxation of uremic vessels to BAY 60–2770, an activator of sGC, that exhibits an optimal effect when the heme group of sGC is oxidized or absent.[26, 31] In accordance, incubation with the very potent oxidizing agent ODQ, increased the sensitivity of control vessels to BAY 60–2770, but not uremic vessels, confirming that sGC is maximally oxidized in CKD.

According to Asif et al.[14], one of the major determinants of the lack of arterial or venous dilation in non-maturating AV fistulas is the poor vascular status of uremic patients. Uremia is potentially linked to endothelial dysfunction as a result of increased oxidative stress. We observed normal endothelial function in uremic vessels, which is in concordance with earlier studies reporting preserved endothelium-dependent relaxation in uremic resistance vessels.[32–34] Decreased endothelium-dependent relaxation, however, has also been reported, caused by an impaired endothelium-derived hyperpolarizing factor (EDHF) response.[35, 36] From our data, it can be concluded that endothelial function is not severely compromised in carotid arteries in CKD, in contrast with SMC function that was clearly diminished due to NO resistance of the NO-receptor sGC. sGC is the soluble form of guanylate cyclase, a widely distributed signal-transduction enzyme that converts GTP into the second messenger cyclic GMP in response to NO and carbon monoxide.[37] It consists of two heterodimers, a larger α-subunit and a heme-binding β-unit, that in its normal Fe^2+^ state acts as the binding site for NO. In case of oxidative stress, sGC can become oxidized resulting in an altered redox state (Fe^3+^) and/or loss of the heme group, after which sGC can no longer be activated by NO, also referred to as NO resistance.[38, 39]

The syndrome of NO resistance can be targeted by a new group of pharmacologic agents including BAY 60–2770, that are possible to either stimulate sGC in a NO-dependent way, or activate sGC in an NO-independent manner. The latter effect is enhanced when sGC exists in its NO-resistant heme-free or oxidized form. NO resistance has also been reported in resistance arteries from diabetes type II patients, hypercholesterolemic rabbits and hypertensive rats.[38] Other potential applications of sGC stimulators/activators such as the treatment of liver fibrosis, pulmonary hypertension and prevention of ischemia-reperfusion injury are currently under investigation.[26, 40, 41]

To investigate a potential beneficial effect of BAY 60–2770 on AV fistula maturation *in vivo*, this compound was administered to CKD animals after creation of the AV fistula. Interestingly, venous diameters increased compared to vehicle-treated CKD animals, but AV fistula flow remained the same. As blood pressure and intima thickness were not significantly different, we cannot explain the lack of increase in flow in BAY 60–2770 treated animals. Although blood pressure was not significantly affected by BAY 60–2770, we cannot exclude the possibility that systemic hemodynamic changes in for instance peripheral resistance or compliance negate the flow effect of an increased diameter at the level of the fistula.

As we were mainly interested in AV fistula maturation our follow-up period encompassed three weeks, in which no difference in neointima formation between CKD and control animals was observed. Other studies with a longer follow-up period reported increased intima thickness in AV fistulas from CKD animals.[23, 24] However, one of these studies was also carried out with a different animal model, using an adenine diet to induce renal failure instead of our nephrectomy model, in which the diet itself could theoretically influence the development of neointima formation.[23] We did observe areas with endothelial denudation, characterized by an eccentric pattern. This was probably related to variations in turbulent flow throughout the fistula. Inadequate outward remodeling, as recently reviewed by Rothuizen et al., can also hamper AV fistula function and is thought to be the result of vascular calcification and insufficient vascular dilatation.[42, 43] Thus, NO resistance with subsequent decreased SMC relaxation could be an important aspect of diminished outward remodeling as well.

In our animals, CKD led to increased systolic blood pressures before and after AV fistula creation. Kokubo et al. reported lower blood pressures in CKD mice compared to controls measured under anesthesia[24], while others did not find any differences with the tail-cuff method.[44] Apparently the effect of CKD on blood pressure differs between animal studies, although in humans renal failure is associated with hypertension[45], which indicates that our animal model accurately reflects the human situation. A potential hypotensive effect of BAY 60–2770 as a result of systemic vasodilation was not observed in our study, but this effect could have been blunted by the presence of the AV fistula. The lack in short-term increase in flow accompanied by larger fistula diameters at the same time needs further investigation. Moreover, it can be of interest to evaluate long-term effects of BAY 60–2770 treatment on neointima formation.

## Conclusion

CKD is associated with decreased AV fistula maturation and NO resistance of sGC in vascular smooth muscle. The sGC-activator BAY 60–2770 can induce vasodilation in CKD that could prevent non-maturation in uremic patients by increasing vessel diameter, although a direct effect on fistula flow was not found in this study.
